# Adnexal Mass Characterization in a Teenage Girl

**DOI:** 10.5334/jbsr.1830

**Published:** 2019-05-10

**Authors:** Denis Danthine, Anne-Fleur La, Laurent Schoysman

**Affiliations:** 1 Department of radiology, CHU Liège, BE; 2 Department of radiology, CHR Citadelle, BE

**Keywords:** adnexal mass, teenager, AFP

A 16-year-old teenager presented to the emergency department for bloating and right-lower-quadrant abdominal pain for two weeks. Mc Burney’s sign was ambiguous, and there was no gynecologic symptom. Blood analysis showed increased C-reactive protein (100 mg/L) and increased LDH (349 U/L) levels.

Ultrasonography revealed ascites and a large hypogastric mass, which appeared to originate from the right ovary. The next morning, contrast-enhanced abdominal computed tomography (Figure 1A, axial view; Figure 1B coronal view; Figure 1C, magnified area-of-interest on 1B) showed an 18-cm heterogeneous right ovarian mass. The core was mostly liquid and the periphery constituted by enhancing tissue and ill-defined cysts of variable sizes. Enlarged intra-tumoral vessels could be distinguished (arrows). There was neither calcification nor fatty component. The massive intraperitoneal fluid was recognized and a greater omentum infiltration was suspected. There was no enlarged lymph node.

**Figure 1:**
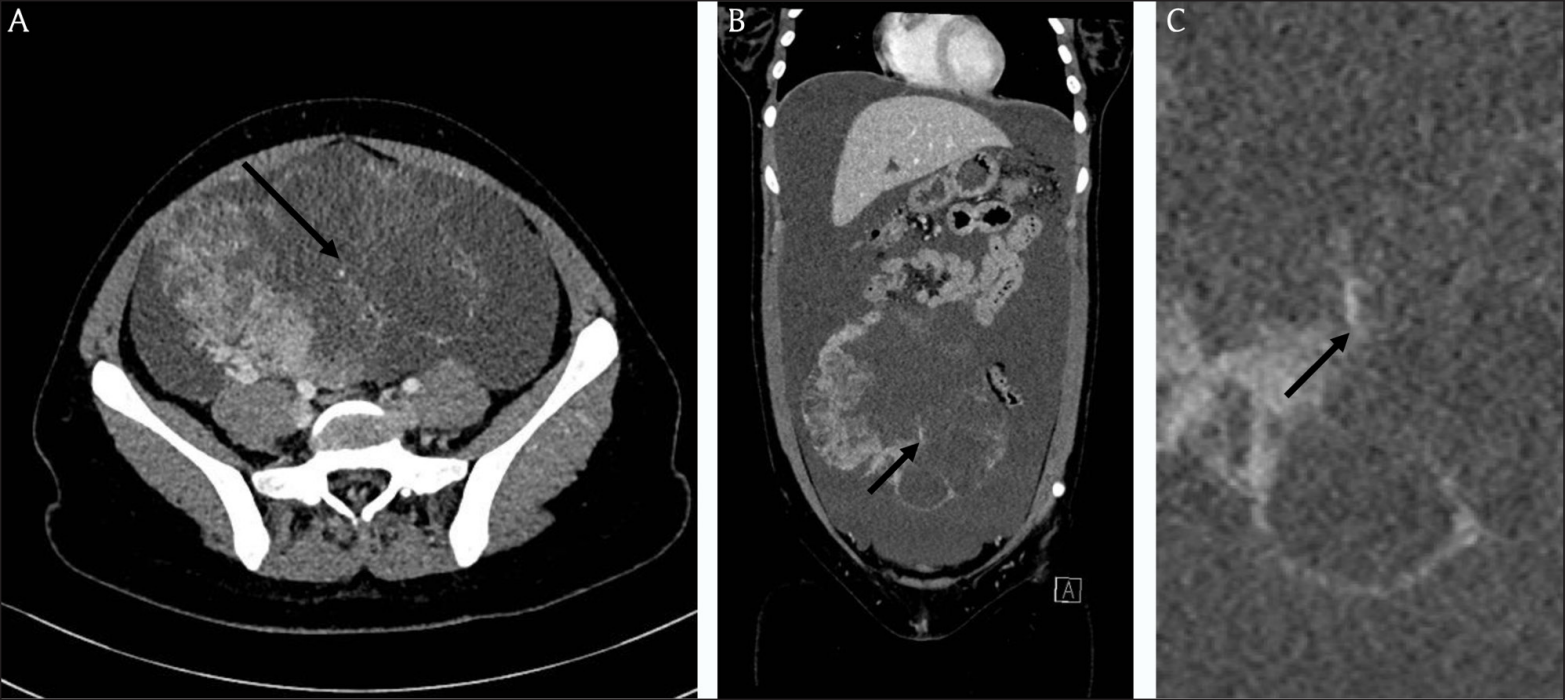


Additional tumor marker tests were performed, showing an elevation of Alpha-foetoprotein (AFP) level to 22,079 microg/L and normal human chorionic gonadotropin beta (beta-HCG). The association of radiological features, patient age, and raised AFP level led to the preoperative diagnosis of yolk sac tumor (YST).

Right adnexectomy and partial omentectomy were carried out by laparotomy and anatomopathological examination confirmed the diagnosis of YST with an intact serous membrane, with mild reactive mesothelial hyperplasia on the omentum.

The patient is currently under close follow-up. Depending on the evolution of AFP level, she might go through chemotherapy or avoid such treatment.

## Comment

Ovarian tumors are rare in children, and 20% of them are malignant, including three main histological types: germinal (80%), stromal (5–10%), and epithelial (10%). YST is the second germinal malignancy after dysgerminoma and accounts for 20% of malignant germ cell tumors (1% of all ovarian cancers).

Radiologists may help to suggest the diagnosis based on radiological features. In this regard, malignancy should be considered when confronted with a large tumor, hyper-vascularization, ascites, or metastasis. There are also more specific features for the diagnosis of YST, such as mixed composition (predominantly cystic), oval shape and well-defined margins, heterogeneous enhancement, and absence of fatty components [1]. Above all these signs, enlarged intra-tumoral vessels is highly suggestive for YST, especially in association with raised blood levels of AFP as in our case [1].
